# Poster Session I - A168 PATIENT-DERIVED ORGANOIDS TO ACCELERATE COLORECTAL CANCER RESEARCH

**DOI:** 10.1093/jcag/gwaf042.168

**Published:** 2026-02-13

**Authors:** S Nassari, M Lecours, J Zhang, F Boudreau

**Affiliations:** Universite de Sherbrooke, Sherbrooke, QC, Canada; Universite de Sherbrooke, Sherbrooke, QC, Canada; Universite de Sherbrooke, Sherbrooke, QC, Canada; Universite de Sherbrooke, Sherbrooke, QC, Canada

## Abstract

**Background:**

In 2024, colorectal cancer (CRC) ranked as the third most frequently diagnosed cancer in Canada and remains one of the leading causes of cancer-related deaths. Current experimental models, including animal models and immortalized cell lines, fail to capture the molecular and cellular heterogeneity of patient tumors. These features are essential for understanding disease mechanisms and developing effective therapies.

Over the past decade, organoid has emerged as a powerful approach to model human tissues in three-dimensional culture while preserving their genetic complexity. In cancer research, patient-derived organoids (PDOs) can be established directly from tumor samples, providing highly relevant models. However, the use of PDOs remains limited by factors such as cost, reproducibility, and restricted access to patient tissues.

**Aims:**

To overcome these challenges, we contributed to the establishment of the Canadian National Organoid Network (CNON), which aims to standardize methods and facilitate access to PDO models for researchers across Canada.

**Methods:**

Within this initiative, the CNON-Sherbrooke node has developed a living biobank of CRC PDOs.

**Results:**

For each patient, tissue samples were collected from both tumor and adjacent normal mucosa to generate paired organoid lines. Up to eight different culture medium formulations were systematically tested. Each condition supporting organoid growth was maintained as an independent autologous tumoroid line. PDOs and their matched primary tissues were processed for whole-genome and transcriptome profiling to assess molecular concordance and validate model fidelity.

With the long-term goal of recapitulating the tumor and its microenvironment, additional autologous tissues were collected, including fibroblasts, PBMCs, and tissue-associated microbiota. Given the critical role of the tumor microenvironment (TME) in cancer development, we implemented an organ-on-a-chip system enabling the co-culture of multiple cell types within a colon-specific chip to better reproduce TME interactions in CRC. Combined with autologous samples, this approach allows the modelling of a patient-specific TME and is expected to (1) improve our understanding of TME involvement in CRC, and (2) enhance drug screening, prognostic assessment, and the development of targeted therapies.

In parallel, we are advancing CRC modeling using genome-editing technologies to reproduce the genetic diversity of CRC and support precision medicine approaches.

**Conclusions:**

In summary, we aim to improve access to standardized CRC PDOs and optimize their culture systems. By integrating organoid biobanking, genome editing, and microfluidic technologies, this initiative will strengthen Canada’s research capacity and contribute to the development of innovative and personalized treatments for colorectal cancer.

**Funding Agencies:**

Weston Foundation Family

